# Acrochordon Chocoe: Characteristics

**Published:** 1881-09

**Authors:** C. F. Nichols

**Affiliations:** Boston


### ACROCHORDON CHOCOE:—CHARACTERISTICS.

C. F. NICHOLS, M. D., BOSTON.

*Verrugosa Acrochordon Chocoe* is a wart-snake of the province of Choco in British Columbia. Symptoms following the bite of the snake, and poisonings from the warts, are detailed in Higgins's *Ophidians.* The following notes are collected from Higgins,* and from notes by Dr. S. Swan. Dr. Swan potentized the gall of the snake (*Fel. Acrochordon Chocoe*), and made provings, which confirmed Higgins's indications.† Higgins reports the gall capable of antidoting the bite, if promptly administered.

*Symptoms.*—Lethargy, tremblings, distortion of the features, loosening of the hair, blood from pores of skin. The bitten limb is thickly covered with small vesicles, filled with an ichorous fluid; when these have attained to the size of a grain of barley they burst, leaving a small sore which increases in size, preserving a funnel shape; they continue sloughing away until they unite one with another, thus destroying all the fleshy substance down to the bone. This is accompanied by intense throbbing pain up the limb. When the poison does not cause death, it yet, almost invariably, produces these *funnel-shaped ulcers.* * * * Death often occurs within three hours. * * * Immediately after the death of the snake, a thick milk-white fluid exudes from the warts, which, applied to the skin of man or beast, produces a well-nigh incurable ulcer. The bile of this snake (ten drops in ten ounces of water) healed, in ten days, a leg, the flesh of which, in consequence of the bite of a Birri snake (called "The Rotter"), had sloughed off up to the knee, leaving the upper surface of the foot and lower leg nearly bare.

Higgins removed the toxical effects of the poison of the *Vipera Calameris Venenosus rubra,* which produces ulcers held to be otherwise

---

* *Ophidians*, pp. 44, 45, 99, 111, 201. † *Advance*, June, 1881, p. 349.

incurable, discharging corrosive yellow ichorous liquid, burning itching, with œdema of the limbs, acute pains in leg, excessive thirst, conjunctival inflammation, lacerating headache, pulse 140–160.

A case of inveterate ulcers of the leg is reported in *Advance*, December, 1880, p. 307, cured by Dr. Nichols with this remedy, CM.

